# Infection Dynamics and Coexistence of Two Novel Arctic Phytoplankton Viruses

**DOI:** 10.3390/v18070726

**Published:** 2026-06-30

**Authors:** Claudia Meyer, Victoria L. N. Jackson, Floris de Haan, Henk Bolhuis, Michael J. Allen, Adam Monier, Corina P. D. Brussaard

**Affiliations:** 1 Department of Marine Microbiology and Biogeochemistry, NIOZ Royal Netherlands Institute for Sea Research, P.O. Box 59, 1790 AB Den Burg, Texel, The Netherlands; 2 Department of Freshwater and Marine Ecology, Institute for Biodiversity and Ecosystem Dynamics (IBED), University of Amsterdam, P.O. Box 94240, 1090 GE Amsterdam, The Netherlands; 3 Living Systems Institute, University of Exeter, Exeter EX4 4QD, UK; 4 Faculty of Health and Life Sciences, University of Exeter, Exeter EX4 4QD, UK

**Keywords:** algal virus, *Phycodnaviridae*, *Micromonas*, virus diversity, coexistence, Arctic Ocean

## Abstract

Marine algal viruses exhibit a high level of diversity, and closely related viruses targeting the same algal host species can stably coexist. Here we report an example of a single virus–host system concealing hidden complexity. We discovered two double stranded (ds) DNA viruses infecting the Arctic picophytoplankter *Micromonas polaris* coexisting in culture for over a decade. Genomic sequencing of the lysate originally characterized as MpoV-44T revealed that it comprises two distinct prasinoviruses with ~203–204 kb genomes (MpoV-44T.A and MpoV-44T.B), of which conserved regions only accounted for 36% (the nucleotide level). The viruses were subsequently separated and compared at both genomic and phenotypic levels. In dual infection studies using a single host strain under nutrient-replete conditions, MpoV-44T.A outcompeted MpoV-44T.B. Yet MpoV-44T.B-like viruses were more abundant than MpoV-44T.A-like ones in natural Arctic metagenomes. This apparent paradox may be explained by differences in host strain specificity and/or possible resilience to nutrient stress by MpoV-44T.B, which we hypothesize based on genomic data. This work unveils hidden virus diversity, illustrating that the dynamics of viral coexistence are not always easily predictable, and underscores the importance of studying the underlying mechanisms at play.

## 1. Introduction

Viruses are significant mortality agents of all phytoplankton taxa [1], with viral lysis-induced loss rates often found to be comparable to those of grazing in phytoplankton communities [2,3,4]. Lytic viruses regulate phytoplankton host population dynamics by lysing host cells, while their host specificity affects phytoplankton succession dynamics and biodiversity [5,6,7,8]. Viral infection may cause phytoplankton bloom decline or even prevent species from blooming in the first place [1,9,10,11]. Consequently, there is a high selection pressure on phytoplankton hosts to develop resistance to viral infection. However, increased resistance to a virus typically comes at cost; it can lead to reduced growth and higher susceptibility to infection by other viruses [12,13,14]. The latter is especially important as most host strains are susceptible to a variety of viruses, just as most virus strains infect more than one host strain [6,10,15,16,17,18,19]. The subsequent virus–host interactions are complex and generate a high degree of intraspecies variation, resulting in stable coexistence of a diversity of viruses and hosts in marine habitats [5,20,21,22,23].

The competitive exclusion principle is a cornerstone of ecological theory; it states that when two species compete for identical resources, one will inevitably outcompete the other [24]. Yet marine viral communities routinely defy this expectation, and closely related viruses targeting the same host species can coexist despite the intense selection pressures imposed by lytic infection cycles [6,15,20,21]. These dynamics can be interpreted through the lens of coexistence theory. This theory divides the mechanisms behind stable species coexistence into stabilizing components, which increase the relative strength of intraspecific competition compared to interspecific competition, and equalizing components, which reduce the average fitness differences between species [25,26,27].

Polar marine virus communities are ecologically distinct from non-polar ones [28,29,30]. In particular, the Arctic Ocean has been shown to harbor high levels of viral diversity with a high proportion of regional and endemic viruses [28,29]. Despite this, few Arctic marine viruses have been isolated and characterized. The Arctic Ocean is arguably one of the environments most affected by climate change, warming up to four times faster than the rest of the globe [31]. Increasing ocean temperatures, combined with an increased freshwater input into the Arctic Ocean, lead to earlier and stronger stratification of Arctic surface waters [32]. Together with the reduction in sea ice cover and increased ocean acidification, these changes are predicted to greatly impact Arctic phytoplankton communities, promoting conditions under which picophytoplankton are expected to thrive [33,34,35]. Furthermore, temperate phytoplankton are forecasted to expand into the Arctic Ocean amidst rising ocean temperatures and increasing inflow of Atlantic waters [36,37,38,39]. Together, these changes have the potential to lead to ecologically relevant shifts in Arctic phytoplankton community composition and function.

Arctic picophytoplankton communities are typically dominated by *Micromonas polaris* (Mamiellophyceae) [40,41,42,43], which occupies the ecological niche typically filled by picocyanobacteria at lower latitudes [40]. *Micromonas* species are susceptible to infection by a large variety of viruses [6,18,44,45]. Thus far, four viruses infecting *Micromonas polaris* have been isolated and brought into culture [19]. These lytic dsDNA prasinoviruses are all specific to *Micromonas* spp. and share many common traits, including a lipid membrane, a capsid diameter of around 120 nm, and a genome size of around 200 kbp [19]. Of the four viruses, MpoV-44T has the broadest host range, infecting both polar and temperate *Micromonas* strains. MpoV-44T has also been reported to be well adapted to higher temperatures, with a shorter latent period and slightly higher burst size at 7 °C compared to 3 °C [19]. These characteristics may position MpoV-44T-like viruses to be better adapted to the rapidly changing conditions of the Arctic Ocean than other MpoV strains. In this study we aimed to characterize MpoV-44T, with our subsequent genomic analysis revealing that MpoV-44T actually comprised two distinct, independently replicating lytic viruses, which we denote as MpoV-44T.A and MpoV-44T.B. Motivated by this, we separated the two viral lineages and combined comparative genomics with controlled infection experiments to further characterize these two viruses. We examined their host ranges as well as infection dynamics within a shared host, revealing their hitherto cryptic coexistence.

## 2. Materials and Methods

### 2.1. Culturing Conditions

Host cultures of *Micromonas polaris* strain RCC2258 (Roscoff Culture Collection) were cultured at 3 °C under around 75 µmol quanta m^−2^ s^−1^ (40FL Panasonic 40SS-ENW/37 lamps, Panasonic, Kadoma, Japan) and a 16:8 h L:D cycle. Cultures were kept in exponential growth phase (maximum growth rate 0.39 d^−1^) by regular transfer (every two weeks) to fresh Mix-TX medium [19]. The *M. polaris* virus MpoV-44T ([19]; NIOZ culture collection) was first isolated in 2006 from Kongsfjorden and maintained since then on *M. polaris* RCC2258 by regular transfer of viral lysate to an exponentially growing host (10% *v*/*v*). MpoV-44T.A and MpoV-44T.B were isolated from MpoV-44T lysate through end-point dilution and maintained separately in the same way as MpoV-44T. For each experiment, fresh viral lysate was produced using an exponentially growing host.

### 2.2. Genome Sequencing

We precipitated viral particles from 50 mL lysate by adding 5 g PEG 8000 and 3.25 g NaCl, mixing thoroughly and incubating on ice at 4 °C overnight. Precipitated viruses were pelleted at 12,000× *g* for 1 h and the supernatant was carefully discarded. We collected the pellet by vigorously rinsing the tubes with 2 × 1 mL sodium chloride/magnesium sulphate (SM) buffer using a P1000 pipette and retaining the resuspended pellet in buffer. DNA was extracted from the resuspended pellet using the Wizard DNA Clean-Up System (Promega, Madison, WI, USA). The sequencing library was prepared for Nanopore (Oxford Nanopore Technologies (ONT), Oxford, UK) sequencing according to the ligation sequencing kit SQK-LSK109 (ONT) and sequenced on a Flongle flow cell (FLO-FLG001; R9.4.1, ONT, Oxford, UK) for 24 h. Basecalling was carried out on the fast5 files with Guppy v3.4.5 using the high-accuracy model (ONT, Oxford, UK).

### 2.3. Genome Assembly and Analysis

Sequencing read quality profiles were examined using FastQC v0.11.9 [46] and used to choose filtering and trimming parameters. Reads were filtered according to their quality scores (min phred score = 11) and length (min length = 350 bp); the first 50 bp were trimmed from the start of the reads using Nanofilt v2.8.0 [47]. Filtered and trimmed reads were checked using FastQC before being taken forward for genome assembly. Genomes were assembled using Flye v2.9-b1768 [48], specifying an input genome size of 200 kbp and using the longest reads with 50× coverage for initial disjointig assembly. The resulting assemblies were polished with the filtered reads using Medaka v1.2.1 (ONT, Oxford, UK) and the r941_min_high_g360 configuration file. CheckV [49] was used to assess genome completeness. Genome annotation was carried out with Prokka v1.14.6 [50] and the–kingdom Viruses parameter. The Prokka output files of predicted protein sequences were used for functional prediction using InterProScan v5.57-90 [51,52]. tRNAs were identified using tRNA-scan-SE v2.0 [53,54].

### 2.4. Comparative Genomic and Synteny Analysis

To assess functional homology and sequence drift between MpoV-44T.A and MpoV-44T.B and their closest relatives, we performed multi-level synteny mapping using pyGenomeViz v1.6.1 [55] across four *Micromonas*-infecting prasinoviruses: Micromonas pusilla virus MpV1, MpoV-44T.A and MpoV-44T.B, and Micromonas commoda virus McV-20T. Protein-level orthologs were identified via reciprocal BLASTp searches across all four genomes and filtered for high confidence using an E-value threshold of <10^−5^, amino acid identity >30%, and a minimum alignment length of 50 residues. For a higher-resolution comparison of the MpoV-44T.A and MpoV-44T.B genomes, nucleotide-level conservation was mapped using BLASTn (BLAST+ v2.16.0). We retained alignments spanning at least 200 bp with >80% identity.

### 2.5. Phylogenetic Reconstruction

Homologous DNA polymerase B (PolB) amino acid sequences from members of the *Phycodnaviridae* were identified using BLASTp searches against the NCBI non-redundant protein database, using PolB sequences from Micromonas pusilla viruses (MpVs) as queries. Retrieved sequences (Supplementary Table S1) were aligned using MAFFT v7.520 [56] with the G-INS-i iterative refinement strategy, suitable for sequences with global homology. The resulting multiple sequence alignment was trimmed using trimAl v1.4 with the -gappyout algorithm to remove poorly aligned or gap-rich positions. Maximum likelihood phylogenetic reconstruction was performed using IQ-TREE v2.2 [57]. ModelFinder implemented in IQ-TREE [58] identified Q.yeast+F+R4 as the best-fitting substitution model according to BIC. Branch support was assessed using 1000 ultrafast bootstrap replicates and the resulting tree was visualized and annotated in iToL [59].

### 2.6. Metagenomic Dataset and Viral Profiling

To investigate the environmental distribution of MpoV-44T.A and -44T.B across the Arctic Ocean, we analyzed metagenomic datasets collected during the Tara Oceans Polar Circle expedition from May to September 2013 [60]. The analysis included 14 samples from 7 distinct sampling stations (Stations 155, 158, 168, 173, 189, 194, and 201), encompassing diverse regions such as the North Atlantic, Norwegian Sea, Kara Sea, Laptev Sea, Chukchi Sea, Beaufort Sea, and Baffin Bay. For each station, samples from two depth layers were examined: the surface layer and the deep chlorophyll maximum (DCM). The taxonomic composition of the viral fraction in these metagenomes was profiled using Kraken2 [61]. The relative abundances (expressed as percentages of total metagenomic sequences) specifically assigned to the distinct viral strains MpoV-44T.A and MpoV-44T.B were extracted for downstream comparative analysis.

### 2.7. Host Range Test

The host range of the newly isolated virus strains, as well as that of the original MpoV-44T lysate, was tested on a range of host strains at different temperatures (Supplementary Table S2) by transferring viral lysate to an exponentially growing host (10% *v*/*v*), mixing regularly and waiting up to 3 weeks to visually confirm lysis. Cultures were considered lysed when they were clear (compared to non-infected host controls).

### 2.8. Infection Experiments

For the virus infection experiments (see for setup also [62]), exponentially growing *M. polaris* host cultures (target concentration of 2 × 10^5^ cells mL^−1^) were infected with freshly produced MpoV-44T (containing both virus lineages in an unknown ratio), MpoV-44T.A only, or MpoV-44T.B at a target virus particle-to-host ratio (v:h) of 10:1 (actual ratios ranged between 8–11:1). We also combined MpoV-44T.A and MpoV-44T.B in equal particle numbers, aiming for a v:h of 10:1 for each virus strain (actual v:h around 7:1). The viruses were added to the host around 3 h into the light cycle. Each treatment was performed in triplicate. Samples were taken directly upon infection (T0) and at regular intervals (typically every 12–24 h until 150 h post infection (p.i.)). As the single virus treatments of MpoV-44T.B appeared to be very slow in their infection dynamics, we repeated the experiment again and sampled the single virus treatment at 408 h post-infection.

Infectivity of MpoV-44T.A and of MpoV-44T.B was determined by end-point dilution followed by Most Probable Number (MPN), as described in [19]. In short, virus lysates were 10-fold diluted with 4.5 mL exponentially growing host culture in 12 dilution steps with 5 replicates for each step and a control row of uninfected host culture. The number of infectious viruses was determined using the MPN Assay Analyzer [63] and divided by the total number of the virus particles (obtained by flow cytometric enumeration; see Section 2.9 below) to obtain the fraction infected viruses.

Infectivity of MpoV-44T.A was around 50% (providing a multiplicity of infection of 4–5 in the infection experiments above), while that of MpoV-44T.B was much lower (around 4% providing and MOI of 0.3). To aim for a MpoV-44T.B infection experiment at MOI > 1 (to ensure one-step infection dynamics) and more comparable to that of MpoV-44T, we repeated the single infection of MpoV-44T.B at a target ratio of 200:1. The actual v:h of 237:1 resulted in a MOI of 9.5, which did not affect the infection dynamics (tested during our pilot experiments).

For phytoplankton and virus enumeration by flow cytometry, two 1 mL samples were fixed with 30 µL formaldehyde:hexamine solution (18% *w/v*:10% *w*/*v*) and with 20 µL 25% glutaraldehyde (EM-grade, Sigma-Aldrich, St. Louis, MO, USA) [64], respectively. Samples were fixed for 15 min at 4 °C, flash frozen in liquid nitrogen and stored at −80 °C until analysis. For qPCR analysis, 1 mL samples were immediately stored at −80 °C until further processing.

### 2.9. Phytoplankton and Virus Enumeration

A benchtop BD Accuri C6 flow cytometer (Becton Dickinson, Franklin Lakes, NJ, USA) equipped with a 488 nm argon laser and the trigger set to chlorophyll red autofluorescence was used to enumerate *Micromonas* cells [65]. A BD FACSCanto flow cytometer (Becton Dickinson, Franklin Lakes, NJ, USA) equipped with a 488 nm argon laser and the trigger on green fluorescence was used to enumerate the stored virus samples ([64] with buffer modification by [66]). The thawed samples were diluted in TE buffer (pH = 8.2, 10 mM Tris-HCl, 1 mM EDTA, filtered through 0.2 µM Minisart NML cellulose acetate filters; Sartorius AG, Goettingen, Germany) and subsequently stained with the nucleic acid-specific green fluorescent dye SYBR-green I (10^−5^ dilution of commercial stock, ThermoFisher Scientific, Waltham, MA, USA) for 10 min at 80 °C in the dark. Afterwards, samples were left to cool (typically 5–15 min) at room temperature in the dark before being analyzed.

FCS express v5 (De Novo Software, Pasadena, CA, USA) was used to analyze the raw data. For phytoplankton, gating was performed on red chlorophyll autofluorescence vs. side scatter and for viruses on green fluorescence vs. side scatter.

From the host and virus abundance plots, we determined the viral latent period (defined as the time taken for the first extracellular viruses appear, considering an increase of at least 10% compared to the preceding measurement), the maximum virus production rates (a linear regression was applied to the period exhibiting the steepest upward slope), and the viral burst size (estimated by dividing the total virus production by the total decrease in phytoplankton host cells).

### 2.10. MpoV Specific qPCR

After discovering that the original MpoV-44T virus lysate contained two distinct virus genomes, we checked our already existing primers for the DNA polymerase B gene (*PolB*) for MpoV-44T on both isolated viruses separately and found that these targeted only MpoV-44T.A. We then designed a range of different primers targeting MpoV-44T.B specifically and selected the best pairs (based on specificity and efficiency). Primers used for MpoV-44T.A were F1 (AACGTCGTTATGGTAACGTT) and R1 (TCATTGATCCTGTTGATGCA) with an annealing temperature of 58.4 °C, and for MpoV-44T.B we used F2 (TTGATATGGCTCCCAAAGAAA) and R2 (GCTGTCCACGTTCAACCAAA) with an annealing temperature of 54 °C. Primer efficiency was assessed through a dilution series of purified PCR product of the respective primers. This dilution series was also used for a calibration curve to allow for quantification of viral *PolB* copies (qPCR, according to [62]). Purified PCR product was produced using fresh viral lysate as a template for a standard PCR, using 5 μL sample added to a 25 μL (total volume) PCR reaction containing 1X AccuStart^TM^ II PCR ToughMix^®^ (Quantabio, LLC., Beverly, MA, USA) and 0.8 µM of each Primer on a BioRad T100 Thermocycler (Bio-Rad Laboratories, Inc., Hercules, CA, USA) with the following program: initial denaturation at 94 °C followed by 40 cycles of 30 s denaturation at 94 °C, 30 s annealing at the designated annealing temperature and 1 min elongation at 72 °C. The PCR product was then loaded onto a 2% agarose gel in 1× TAE buffer (40 mM Tris, 21 mM acetic acid, 1 mM EDTA), stained with 0.25× SYBR Safe (Invitrogen, Waltham, MA, USA) and run at 80 V for around 85 min. The product bands were cut out under blue light, purified using the QIAquick Gel Extraction Kit (Qiagen, Hilden, Germany), and measured using the Qubit HS DNA assay on a Qubit 3.0 fluorometer (Invitrogen, Waltham, MA, USA). Subsequently, a dilution series was set up in Tris buffer, with a targeted concentration range of 10^9^ down to 10 copies per µL. The different dilutions were immediately aliquoted and stored at −80 °C.

For analysis of experimental samples, the frozen, untreated qPCR samples from the experiments were slowly thawed at 4 °C and diluted with 4 mL of cold ultrapure water (19.2 Ω cm^−2^). Samples were treated by sonication, with a program of 3 intervals at an amplitude of ~8 for 10 s and a break of 30 s in between using an MSE Soniprep 150 Ultrasonic disintegrator (MSE Ltd., London, UK). Samples were constantly kept on ice during treatment and stored at −80 °C until use. The cellular and cell-free fractions were treated as a whole and not separated.

For qPCR analysis, the Bio-Rad CFX96 Touch Real-Time PCR Detection System (Bio-Rad Laboratories, Inc., Hercules, CA, USA) was used. qPCR reactions contained 5 μL of sonicated sample, 0.8 µM of each Primer, 1× EvaGreen Dye (Biotium, Fremont, CA, USA), and 1× AccuStart II PCR ToughMix^®^ (Quantabio, Beverly, MA, USA), in a total volume of 25 μL. The qPCR program contained an initial denaturation step at 94 °C followed by 40 cycles of 30 s denaturation at 94 °C, 30 s annealing at the designated annealing temperature and 1 min elongation at 72 °C. After amplification, a melting curve from 65 °C to 95 °C, with 0.5 °C increments, was performed to assess product length. Non-template controls containing ultrapure water instead of sample, as well as off-target controls containing samples with the non-targeted viruses, were included to control for primer specificity. A calibration curve based on triplicates of the dilution series was used for amplicon quantification in the samples, as well as for assessing primer efficiencies. Primer efficiencies for the qPCRs were 93.1% and 93.8% for the MpoV-44T.A primer pair and 83.2% and 89.1% for the MpoV-44T.B primer pair.

For qPCR data analysis, maximum genome production rates were calculated based on a linear regression applied to the period exhibiting the steepest upward slope rates. For comparison between different qPCR sets, especially different primer pairs and separate experiments, results were min–max normalized according to Equation (1),

x′ = (x − min(X))/(max(X) − min(X))(1)

with x′ being the normalized value, x the original value, and min(X) and max(X) the minima and maxima of the averaged qPCR results from the single virus treatment of the targeted virus strain.

### 2.11. Screening of Archived MpoV-44T Lysates

We screened archived MpoV-44T lysates going back to 2009 for the presence of both MpoV-44T strains. These lysates were all generated using the original MpoV-44T isolate [19] and were produced by adding 0.5 mL of virus lysate to 4.5 mL of exponentially growing host. Lysis usually took around 1 week, after which the lysates were stored at 4 °C, with monthly maintenance intervals. A 1 mL sample from lysates stored at 4 °C was treated with 4 mL of cold Ultrapure water (19.2 Ω cm^−2^) and immediately used for qPCR (without a calibration curve; qPCR was only used instead of PCR to allow for better discrimination between positive and negative samples). Samples of the targeted strain were included as a positive control, while samples of the non-targeted MpoV-44T strain were included as a negative control. Samples were considered positive when their Cq (quantification cycle) value was >30, with no negative controls reaching that threshold.

### 2.12. Statistical Analysis

R version 4.2.1 was used for statistical analysis. For parametric testing, either a *t*-test or ANOVA (followed by a Tukey HSD post hoc test) was employed. Prior to parametric testing, a Levene’s test was used to check for homogeneity of variances. If variances were non-homogeneous, non-parametric tests were used: a Wilcoxon rank sum test or a Kruskal–Wallis rank sum test (followed by a Dunn’s test of multiple comparisons).

## 3. Results

### 3.1. Two Distinct Virus Strains

**Figure 1:**
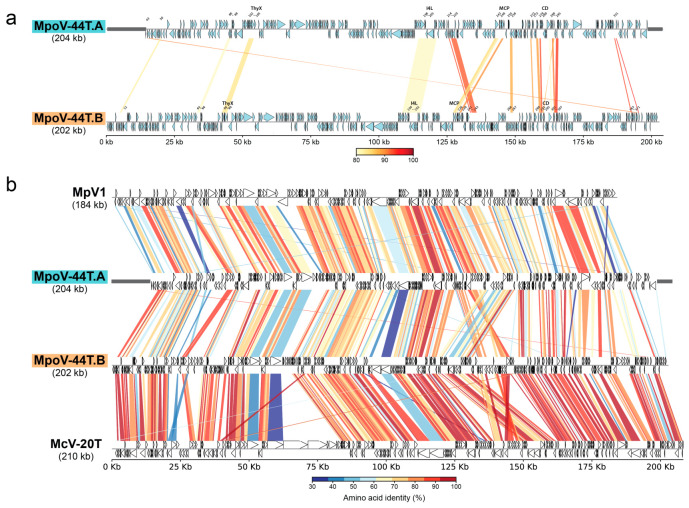
Conserved genomic structure and synteny across Micromonas viruses. (**a**) Nucleotide sequence collinearity between MpoV-44T.A and MpoV-44T.B. The linear genomic map depicts predicted native ORFs as light blue arrows. Solid shaded ribbons spanning across the two genome tracks represent regions of orthologous nucleotide homology identified by BLASTn. The ribbon shading reflects the continuous degree of nucleotide identity (from 80 to 100%; see scale bar). Select genes located in these highly conserved blocks are labeled above their respective genomic coordinates: ThyX (thymidylate synthase), I4L (ribonucleoside-diphosphate reductase large subunit), MCP (major capsid protein), CD (deoxycytidylate deaminase); identifiers correspond to gene sequence identifiers. (**b**) Four-way protein-level sequence conservation comparing the genomes of MpV1, MpoV-44T.A, MpoV-44T.B, and McV-20T. Ribbons connecting contiguous genomic tracks define homologous protein sequences mapped via reciprocal best hits from BLASTp. The ribbon shading reflects the continuous degree of amino acid identity (from 30 to 100%; see scale bar). In both panels, arrows represent predicted protein-coding genes, and their orientation indicates the coding strand (rightward for the forward strand and leftward for the reverse strand).

Genome sequencing of the MpoV-44T lysate resolved two distinct viral genomes of 204,290 bp (MpoV-44T.A) and 202,769 bp (MpoV-44T.B), with GC contents of 37.48% and 39.54% and 363 and 383 predicted CDSs (coding DNA sequences), respectively. Depth of coverage was high (1324× and 1552×), providing strong evidence in support of two distinct genomes. Genome completeness, assessed using CheckV, was determined to be 100% for both MpoVs. The genomes shared just 36% in conserved areas on the nucleotide level (Figure 1a) and 70.38% on the amino acid level (Figure 1b). The highest level of amino acid similarity between MpoV-44T.A and another virus was with MpV1, sharing 69.94% similarity. For MpoV-44T.B, the highest similarity was with McV-20T, sharing 84.41% similarity. Notably, the termini of MpoV-44T.A formed inverted terminal repeats (ITRs) built from a tandem ~333 bp unit, present at both ends in inverted orientation but differing in copy number (5′: 15,184 bp/~45 units; 3′: 5694 bp/~17 units), with the 3′ array representing a subset of the 5′ one; the asymmetry therefore reflects copy-number variation rather than incomplete assembly. The repeats are AT-rich (31% GC) and longer than the ITRs reported for other prasinoviruses [67]. No significant terminal repeats were detected in MpoV-44T.B, and the ITRs are not conserved between the two strains. Orthology analysis shows large accessory repertoires in both MpoV-44T genomes: 44T.B encodes, among others, two FkbM methyltransferases, a glycerophosphodiester phosphodiesterase (GP-PDE), and an aspartyl/asparaginyl β-hydroxylase, whereas 44T.A encodes enzymes including phosphoglycolate phosphatase and 3-dehydroquinate synthase, plus a betaine/carnitine/choline (BCCT)-family transporter.

**Figure 2:**
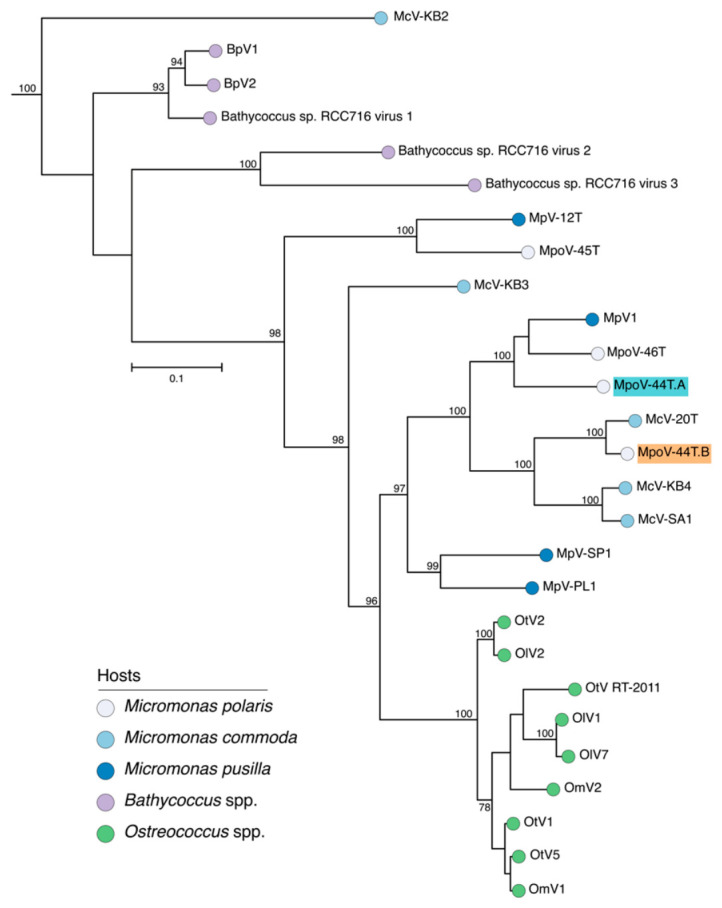
Maximum-likelihood phylogeny of prasinovirus DNA polymerase B (PolB) protein sequences, inferred from full-length PolB proteins with IQ-TREE 2 under the Q.yeast+F+R4 substitution model, selected by ModelFinder (BIC). The alignment comprised 32 sequences and 940 amino acid positions. Branch support was assessed with 1000 ultrafast bootstrap replicates; values ≥75 are shown at nodes. MpoV-44T.A (orange) and MpoV-44T.B (aqua) occupy distinct phylogenetic positions: MpoV-44T.A is sister to a clade comprising MpV1 and MpoV-46T (100% bootstrap), whereas MpoV-44T.B is sister to Micromonas commoda virus McV-20T (100% bootstrap), nested within a broader clade of M. commoda viruses (McVs). The third M. polaris virus, MpoV-45T, is sister to MpV-12T (100% bootstrap). The tree is rooted on chloroviruses (4 PbCVs, AtCV-1; not shown); the scale bar represents substitutions per site. Accession numbers for sequences used in this phylogeny are available in Supplementary Table S1.

Phylogenetic analysis of DNA polymerase B (PolB) further confirmed that MpoV-44T.A and MpoV-44T.B are distinct viruses rather than variants of a single lineage. In the PolB maximum likelihood tree (Figure 2), MpoV-44T.A clustered with MpV-1, while MpoV-44T.B clustered with McV-20T.

Despite their apparent cryptic coexistence since initial isolation, we were able to separate, isolate and maintain MpoV-44T.A and MpoV-44T.B, indicating that they are not dependent on each other for successful infection. A host range test (Supplementary Table S2) revealed that the two strains could infect Arctic as well as temperate *Micromonas* strains (*M. polaris*, *pusilla* and *commoda*). While they mostly overlapped in their host range, MpoV-44T.A was able to infect one strain of *Micromonas polaris* (RCC2242) that MpoV-44T.B could not. Conversely, MpoV-44T.B was able to infect *M. pusilla* strain LAC38 at 15 °C, while MpoV-44T.A was not, despite both being able to infect this strain at 7 °C.

### 3.2. Single Virus Infection Dynamics

**Figure 3:**
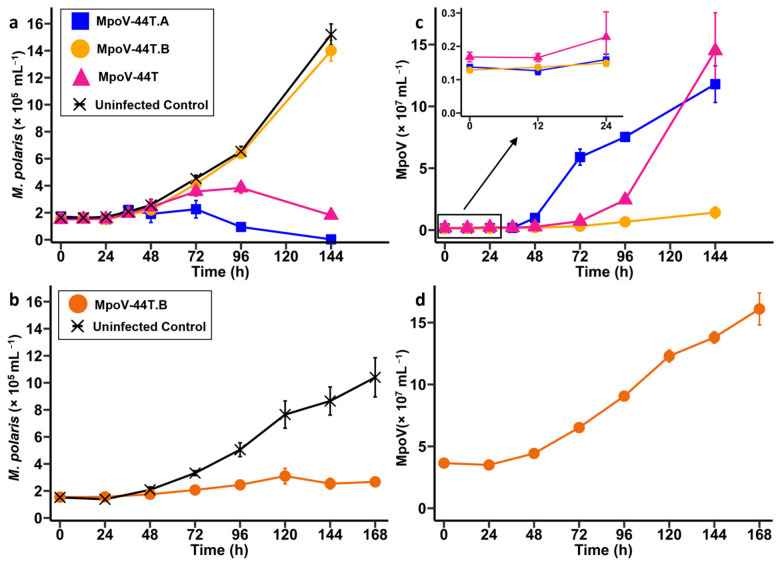
Temporal dynamics of *Micromonas polaris* (**a**,**b**) uninfected (black cross) and infected with viruses MpoV-44T ((**c**); pink triangle), MpoV-44T.A ((**c**); blue square) or MpoV-44T.B ((**c**,**d**); orange circle). Inset in panel (**c**) shows the first 24 h p.i with MpoV-44T.A, MpoV-44T.B or MpoV-44T to highlight the latent period. Initial experiment (**a**,**c**) shows infection with a target virus:host (v:h) ratio of 10:1. Since infectivity of MpoV-44T.B turned out to be very low (4%), we repeated the infection experiment with a target v:h of 200:1 (**b**,**d**). Results are shown as averages ± standard (*n* = 3).

The infection dynamics of each of the isolated virus strains were examined using *Micromonas polaris* RCC2258 as host and an initial target v:h of 10:1. MpoV-44T.A infection resulted in host growth plateauing at 36 h p.i., with host cell lysis starting at 72 h p.i. and lasting for another 72 h until full lysis of the culture (Figure 3a). In contrast, infection with MpoV-44T.B did not result in lysis within the 6-day duration of the experiment (Figure 3a). When repeating the MpoV-44T.B infection experiment for a longer duration, host growth plateaued after 200 h p.i. and the culture collapsed about 240 h p.i. (Figure S1a). Due to the low infectivity of MpoV-44T.B (4%), another infection experiment was performed with an increased v:h, resulting in an MOI of 9.5 (compared to 0.3 in the v:h of 10:1, and around 4–5 for MpoV-44T.A). Growth of the host was strongly reduced (Figure 3c). Still, the host lysis dynamics in this experiment did not compare to the more rapid lysis of *M. polaris* after infection with MpoV-44T.A (Figure 3a,b). Growth of the host was, however, strongly reduced (Figure 3c).

Both MpoV-44T.A and MpoV-44T.B had a latent period of 12–24 h, with maximum virus progeny production rates starting at around 36–48 h p.i. for MpoV-44T.A and at 48 h p.i. for MpoV-44T.B (Figure 3c,d). Virus production rate was 1.4-fold higher for MpoV-44T.A (1.52 ± 0.27 × 10^6^ vs. 1.09 ± 0.04 × 10^6^ particles mL^−1^ h^−1^, respectively), albeit not significantly. Viral burst sizes were 488 ± 101 per lysed host cell for MpoV-44T.A and 581 ± 188 per lysed host cell for MpoV-44T.B. We cannot rule out that there was some level of continuous growth and lysis of the host cells and therefore the burst size of MpoV-44T.B may have been overestimated.

### 3.3. Coexistence

Upon the discovery of the two different MpoV strains, we screened for the presence of both virus strains in archived MpoV-44T lysates that had been stored at 4 °C. We detected both viruses in lysate dating back as far as 2009 (soon after isolation of MpoV-44T; Supplementary Table S3), indicating these viruses have coexisted in alternating culture and storage most likely since their initial isolation. The infection dynamics in the MpoV-44T treatment (which contained both MpoV-44T.A and MpoV-44T.B in an unknown ratio, v:h of 10:1) mostly resembled that of MpoV-44T.A, but with a delay of about 24 h in both host lysis (started after 96 h p.i.; Figure 3a) and maximum virus production (starting at 72 h p.i., Figure 3c). The latent period (12–24 h) was similar to both the single viruses, and the maximum virus production rate and burst size (1.46 × ±0.98 × 10^6^ particles mL^−1^ h^−1^ and 576 per lysed host cell) were comparable to MpoV-44T.A.

**Figure 4:**
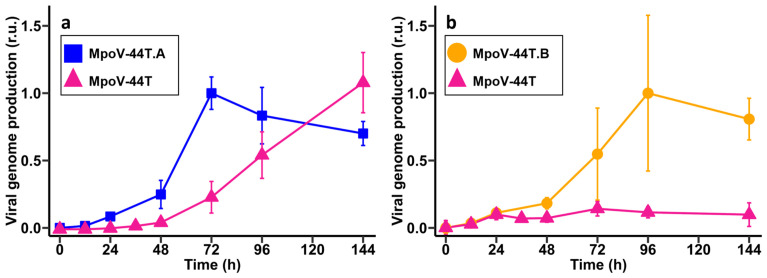
Viral genome production of MpoV-44T.A (**a**) and MpoV-44T.B (**b**) in single infection or in MpoV-44T. Viral genome abundances are min–max normalized with the respective single virus infection treatment as reference. Results are shown as averages ± standard deviation (*n* = 3).

Since we could not differentiate between MpoV-44T.A and MpoV-44T.B particles by flow cytometry, we also performed qPCR assays for the experiment displayed in Figure 3a,c to assess how each virus performed in the MpoV-44T infection treatment, using genome production as a proxy (Figure 4). Genome production of MpoV-44T.A reached similar levels in the single MpoV-44T.A and MpoV-44T infection treatments (4.94 ± 0.74 × 10^7^ and 5.23 ± 1.07 × 10^7^ *PolB* copies mL^−1^, respectively; Figure 4a). Maximum genome production rates of MpoV-44T.A were similar (7.72 ± 2.33 × 10^5^ *PolB* copies h^−1^ and 5.51 ± 0.88 × 10^5^ *PolB* copies h^−1^, respectively) but were delayed by 24 h in MpoV-44T (similarly to the virus particle enumeration results). In contrast, while MpoV-44T.B genome replication rates were comparable in single and MpoV-44T infection in the first 24 h, they plateaued in the MpoV-44T infection while still increasing in the single MpoV-44T.B infection after 24 h (Figure 4b). In total, MpoV-44T.B genome production was significantly suppressed in the MpoV-44T treatment, with only about 14 ± 1% of the single MpoV-44T.B infection produced (9.7 ± 0.9 × 10^5^ vs. 7.1 ± 1.6 × 10^6^ *PolB* copies mL^−1^; Student’s *t*-test, t(4) = 6.7, *p* = 0.003).

The delayed MpoV-44T.A genome production in the MpoV-44T infection treatment compared to infection with MpoV-44T.A only was most likely due to the lower abundances of MpoV-44T.A in the MpoV-44T lysate. The number of MpoV-44T.A genome copies at the start (T0) of the infection experiment was substantially lower for the MpoV-44T infection treatment than for the single MpoV-44T.A infection (0.6 ± 0.5 × 10^5^ vs. 5.4 ± 0.9 × 10^5^ *PolB* copies mL^−1^), while the number of genome copies of MpoV-44T.B was comparable between the MpoV-44T infection treatment and the respective single infection (5.3 ± 3.2 × 10^5^ and 5.3 ± 2.4 × 10^5^ *PolB* copies mL^−1^).

**Figure 5:**
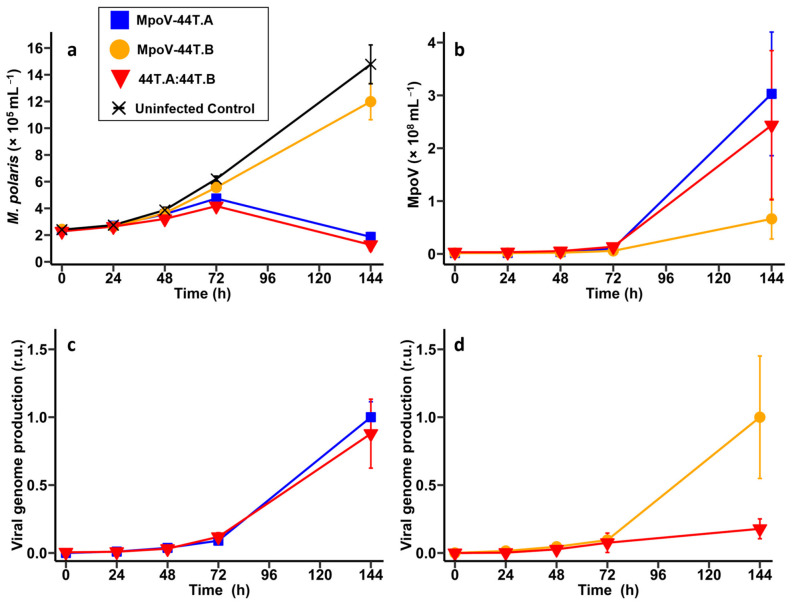
Dual infection dynamics of MpoV-44T.A and 44T.B compared to single infections (v:h 10:1 for each virus). (**a**) *Micromonas polaris* and (**b**) MpoV particle dynamics obtained by flow cytometry. (**c**,**d**) Viral genome production of MpoV-44T.A (**c**) and MpoV-44T.B (**d**). Viral genome abundances are min–max normalized with the respective single virus infection treatment as reference. Results are shown as averages ± standard deviation (*n* = 3).

We thus performed a dual infection experiment with a target v:h ratio of 10:1 for each virus (MOI = 3.5 and 0.3 for MpoV-44T.A and MpoV-44T.B, respectively), to test if and how MpoV-44T.A would be affected by the presence of MpoV-44T.B when both viruses were present in equal numbers (dual infection treatment, Figure 5). This treatment displayed similar host lysis and virus particle production dynamics (Figure 5a,b) as the MpoV-44T.A single treatment, with maximum production rates of 2.65 ± 1.59 × 10^6^ and 3.34 ± 1.31 × 10^6^ particles mL^−1^ h^−1^, respectively. Furthermore, no significant changes in genome production (rate and total) were observed for MpoV-44T.A in the dual compared to the single infection (Figure 5c). In contrast, the genome production of MpoV-44T.B was significantly suppressed in the dual infection treatment (Figure 5d) and reached only 18 ± 8% of the single infection treatment (8.0 ± 3.4 × 10^6^ vs. 4.4 ± 2.0 × 10^7^ *PolB* copies mL^−1^: Student’s *t*-test t(4) = -3.07, *p* = 0.037).

### 3.4. MpoV-44T.A and MpoV-44T.B Biogeography

**Figure 6:**
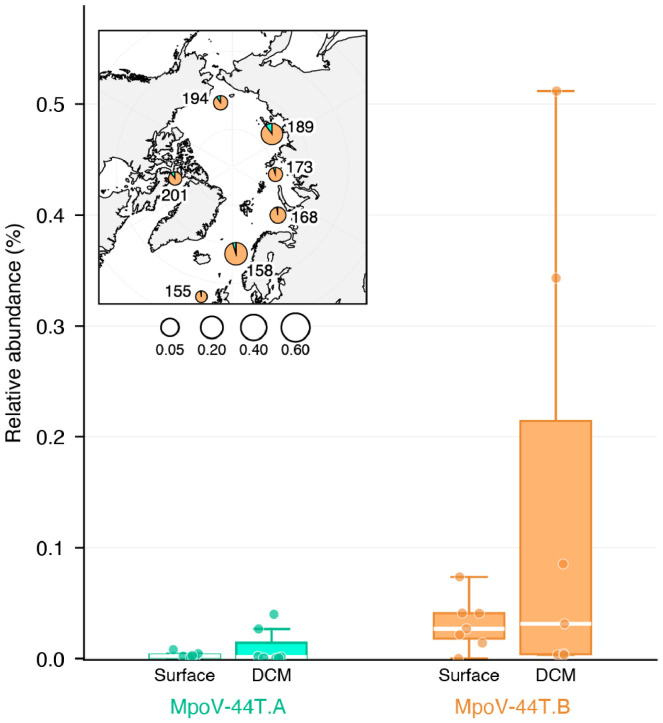
Biogeographic distribution and relative abundance of MpoV-44T.A and MpoV-44T.B in Arctic Ocean metagenomes. Boxplots show the relative abundance (% of total metagenomic reads) of MpoV-44T.A (aqua) and MpoV-44T.B (orange) at surface and deep chlorophyll maximum (DCM) depths across seven Tara Oceans Polar Circle stations. Individual data points are overlaid for each station. MpoV-44T.B was consistently more abundant than MpoV-44T.A at both depths, with highest values at the DCM. Inset: North Polar map showing the relative proportions of MpoV-44T.A and MpoV-44T.B at each sampling station. Pie chart area is proportional to the total combined abundance of both viruses (summed across depths); at the North Atlantic station (155), both strains occur only at trace levels, shown as a correspondingly small chart.

Metagenomic read classification of Tara Oceans Polar Circle stations (Stations 155, 158, 168, 173, 189, 194, and 201; sampled between May and September 2013) with paired surface and deep chlorophyll maximum (DCM) samples recovered a consistent biogeographic signal for the two MpoV-44T strains (Figure 6). At the southernmost North Atlantic station (Station 155), both strains were present only at trace levels (combined relative abundance 0.004% of total metagenomic reads), roughly two orders of magnitude lower than at the Arctic stations. Within the Arctic stations, genotype composition was consistently dominated by MpoV-44T.B (mean relative abundance 0.086% of total metagenomic reads), which was present at significantly higher levels than MpoV-44T.A (mean relative abundance 0.006%) across the sampled sites (Wilcoxon signed-rank test, *p* = 1.2 × 10^−4^).

## 4. Discussion

### 4.1. Two Distinct Virus Strains Coexisting in Culture

Our data show that the virus lysate originally characterized as MpoV-44T [19] has, likely since isolation, been a covert mixture of two independently replicating viruses. High-coverage genomes, strain-specific qPCR, and retrospective screening of archived lysates demonstrate that MpoV-44T.A and MpoV-44T.B coexisted in culture for >14 years. These two closely related lytic dsDNA prasinoviruses with ~203–204 kb genomes were distinct enough in their genomes to show that MpoV-44T.A and MpoV-44T.B were not merely variations in one another generated during laboratory domestication. Indeed, comparative genomic synteny revealed that while they share broad structural collinearity across core machinery (e.g., major capsid proteins), their overall average amino acid identity across orthologs is only ~70%. Furthermore, nucleotide-level conservation was limited to ~36% of their aligned genomes, represented by fractured syntenic blocks rather than contiguous homologous sequences. This large sequence divergence, which is comparable to the genetic distances separating them from distinct Micromonas virus isolates such as MpV1 and McV-20T, indicates that these strains diverged substantially in the environment well prior to their co-isolation. This divergence is further evidenced by the DNA polymerase B phylogeny, which places MpoV-44T.A and MpoV-44T.B onto distinct clades; MpoV-44T.A clusters with MpV1, whereas MpoV-44T.B groups separately as a sister taxon to McV-20T, definitively confirming their status as distinct environmental viruses rather than recent laboratory variants. While they had comparable latent periods to other MpoVs and MpVs, they both had relatively high burst sizes (higher than reported for other MpoVs, and on the higher end of typical MpV burst sizes [6,19,62]).

Despite their coexistence in culture, MpoV-44T.B was outcompeted by MpoV-44T.A in infection experiments using the original MpoV-44T lysate, as well as when adding both viruses in equal (particle) numbers to *M. polaris* cultures. Virus–virus interactions, including negative interference, can occur at different stages of the infection cycle. Viruses infecting a cell may prevent other viruses from infecting the same cell, a process known as superinfection exclusion [68,69]. Viruses may also interfere with each other when infecting the same cell, as was recently described for two other MpoVs [62]. Such virus competition and coexistence may result in recombination of both viruses, generating new viral strains and increasing viral diversity. If the viruses were indeed coinfecting the same cell, the ITR size variation between MpoV-44T.A and MpoV-44T.B may have played a role in modulating their competitive interactions. Long ITRs have been associated with functions in genome replication, viral packaging, and DNA recombination [70], and were speculated to play a role in competitive interactions between two other MpoVs, MpoV-45T and MpoV-46T [62]. Here, the ITRs of the dominant MpoV-45T were substantially longer than those of MpoV-46T. Similarly, MpoV-44T.A’s longer ITRs could have given it a competitive advantage over MpoV-44T.B. Further research (e.g., transcriptomics) is needed to determine if coinfection of the same host cell occurs and to determine in more detail which factors are underlying these mechanisms and driving the dynamics reported here. Still, our study demonstrates that different viruses may easily be propagated together in culture, suggesting stable viral coexistence may be more common than previously thought.

The precise mechanisms behind the phenotypic differences displayed by MpoV-44T.A and MpoV-44T.B are difficult to determine at this point in time. Nevertheless, their genomes do provide some clues on which we can speculate. A unique feature of the MpoV-44T.A genome is the presence of a transmembrane transporter protein belonging to the BCCT family, potentially originating from a host-to-virus horizontal gene transfer (HGT) [71]. BCCTs are primarily known for their role in transporting compatible solutes essential for metabolism, osmoregulation, and stress responses [71,72]. We speculate that their function in osmoregulation [71] may mean that MpoV-44T.A could influence host cell lysis and the release of viral progeny. The discovery of this putative HGT presents an opportunity for future research to work on characterizing this protein, from its phylogenetic origin and molecular structure to its transcriptional activity and function in infection, as has previously been done for a host-derived ammonium transporter in an Ostreococcus tauri virus [73].

### 4.2. Persistence of MpoV-44T.B

Although we do not know the exact amount of both virus strains in the MpoV-44T lysate, we did observe that there were substantially fewer genome copies of MpoV-44T.A at the start of the MpoV-44T infection experiment than in the single MpoV-44T.A infection, while there were roughly equal genome copies of MpoV-44T.B in both MpoV-44T and 44T.B-only infection. Consequently, if MpoV-44T.A was present in lower numbers in MpoV-44T, it may have needed more than one infection cycle to infect the majority of host cells, resulting in the observed delay. When the viruses were added in equal numbers (based on virus particle counts), MpoV-44T.A was clearly dominant, further supporting the hypothesis that the original MpoV-44T lysate was made up of more MpoV-44T.B particles than MpoV-44T.A particles. Higher viral decay rates of MpoV-44T.A may have contributed to relatively higher abundances of MpoV-44T.B particles, but we did not find any significant changes in virus counts upon storage at 4 °C. Specific higher loss of infectivity of MpoV-44T.A was also not noted.

Consistent (longer-term) coexistence of MpoV-44T.B may have been partly due to the relatively high inoculate volume (10% *v*/*v*) used when maintaining viral lysates in culture, and consequently high v:h ratios used during maintenance of MpoV-44T in culture. However, considering the much lower infectivity of MpoV-44T.B (compared to MpoV-44T.A), it seems unlikely that this alone would have been enough to facilitate persistence of MpoV-44T.B. MpoV-44T.B would have had to consistently produce more virus particles to keep up. Although the burst size of MpoV-44T.B seemed higher than that of MpoV-44T.A, this difference would likely not be enough to offset its low infectivity. According to the competitive exclusion principle [24], MpoV-44T.B should have been outcompeted by MpoV-44T.A. A possible explanation of the virus’ continued persistence might be given through coexistence theory, which predicts that stable coexistence requires niche differences to exceed fitness differences between competitors [25,26,27]. These niche differences may have been provided through a complex interplay of host and virus microdiversity and microevolutionary processes. For example, heterogeneity in host cultures (polyclonality) could have led to differences in susceptibility to the viruses, either in subpopulations of the same host culture or over time. Additionally, trade-offs in virus evolution, for example, between growth and survival [74], could have helped MpoV-44T.B persist.

### 4.3. Distribution of MpoV-44T in Arctic Metagenomes

Both strains were detected in Arctic metagenomes, with MpoV-44T.B being significantly more abundant. The dominance of MpoV-44T.B in polar metagenomes contrasts with its pronounced suppression during dual infections in vitro, pointing to niche partitioning and/or fluctuating selection pressures in nature. Concurrently, *M. polaris* clade II was more abundant than clade I in these same samples [39]. Clade II *M. polaris* is typically more abundant in subsurface samples [39]. In the subsurface chlorophyll maxima (SCM) characteristic of the stratified Arctic Ocean in summer, light levels can be <10% of those at the surface [75], and *M. polaris* occupying this niche may grow relatively slower and take longer to reach high cell densities [41]. Perhaps, the more ‘chronic’ infections of MpoV-44T.B observed in vitro and resulting in slow host lysis are an adaptation to infecting slower growing host. It seems likely that MpoV-44T.B’s dominance in the environmental samples may be due to better adaptation to the clade II host strains in these samples. A study on *M. pusilla* viruses showed that MpV infection was clade-specific and, depending on the infected clade, different infection strategies were displayed [6]. Assuming this a general feature for Micromonas viruses, MpoV-44T.B’s infection strategy could be more tailored to a different host ecotype, and using an *M. polaris* clade I strain for our experiments may have been sub-optimal for MpoV-44T.B.

Better adaption to higher temperatures could be another factor in MpoV-44T.B’s environmental dominance over MpoV-44T.A. Only MpoV-44T.B was capable of host lysis at 15 °C, suggesting the previous observation of MpoV-44T being the only MpoV isolate capable of host lysis at 15 and 20 °C [19] was driven by MpoV-44T.B. Given the rapid warming of the Arctic Ocean (AO) [31], this temperature tolerance, along with its ability to infect temperate *Micromonas* hosts, which are predicted to expand into the AO [36,37,38,39], would suggest MpoV-44T.B to be well adapted to future AO conditions.

Additionally, accessory-gene differences (e.g., BCCT in MpoV-44T.A; GP-PDE in MpoV-44T.B) may provide mechanistic hypotheses for differential fitness across host physiology and environmental regimes. Bacterial GP-PDEs liberate phosphate from membrane glycerophosphodiesters under P-stress [76]. Viral auxiliary metabolic genes (AMGs) related to P-metabolism can provide a selective advantage under P-limitation [77]. P-limitation constrains Micromonas virus proliferation [78,79], and some prasinoviruses have been reported to encode the phosphate transporter PHO4 [80]. A virus encoding a GP-PDE, such as MpoV-44T.B, might therefore be better able to scavenge phosphate from its host during infection under the P-limited conditions typical of summer Arctic surface waters [81,82,83]. Since the Tara Oceans samples used in this study were collected during summer (between May and September), we cannot exclude seasonal effects on the viruses’ distribution. MpoV-44T was originally isolated in December of 2006, and it is possible that MpoV-44T.A is better adapted to winter conditions, either due to seasonal variabilities in host composition or better adaption to environmental conditions.

Together, our results show that virus diversity can be easily overlooked, and that analyzing the genomes of virus isolates is crucial to detecting hidden diversity. In addition, we underline that virus competition and/or coexistence can be difficult to predict, and may depend on a complex variety of factors, including genotypic and phenotypic properties of viruses as well as of their hosts, alongside abiotic environmental factors such as temperature and nutrient limitation.

## Data Availability

All flow cytometry and qPCR data used in this publication are publicly available at https://doi.org/10.25850/nioz/7b.b.vk. The MpoV genomic sequencing reads and genome assemblies have been deposited under NCBI BioProject PRJNA1443695.

## Supplementary Materials

The following supporting information can be downloaded at: https://www.mdpi.com/article/10.3390/v18070726/s1, Table S1. Accession numbers of viral PolB sequences retrieved from NCBI BLASTp searches and used for phylogenetic reconstruction; Table S2: Host range test of MpoV-44T.A, MpoV-44T.B and MpoV-44T lysate on 11 different Micromonas polaris and Micromonas pusilla strains at four different temperatures; Figure S1. Infection dynamics of MpoV-44T.B over the course of 408 h (17 days); Table S3: Screening of archived MpoV-44T lysates for presence of MpoV-44T.A and 44T.B using qPCR.
