# Supplementary material for: Infection Dynamics and Coexistence of Two Novel Arctic Phytoplankton Viruses
Source: Viruses. 2026 Jun 30;18(7):726. doi: 10.3390/v18070726 (PMC13431477; doi:10.3390/v18070726)
Supplement: Supplementary file 1 [file viruses-18-00726-s001.zip › viruses-4262594-supplementary.pdf]

Table S1. Accession numbers of viral PolB sequences retrieved from NCBI BLASTp searches and used for phylogenetic reconstruction in Fig. 2.

Virus	NCBI accession
OtV1	YP_003213031
OtV2	YP_004063640
OtV5	YP_001648316
OtV-RT-2011	AFC35136
OlV1	YP_004061851
OlV2	ALI95603
OlV7	YP_009173227
OmV1	YP_009172985
OmV2	QIZ31260
BpV1	YP_004061614
BpV2	ADQ91356
BpV-RCC716-1	QOR60232
BpV-RCC716-2	QOR60350
BpV-RCC716-3	QOR60731
MpV1	YP_004062103
MpV-SP1	YP_009665020
MpV-12T	YP_007676285
MpV-PL1	AET43521
McV-20T	XKM46652
McV-KB2	XCA47438
McV-KB3	XDR79888
McV-KB4	XHR79304
McV-SA1	XIF71115
MpoV-45T	genome: PP728250
MpoV-46T	XCJ77185 (genome: PP728251)
PbCV-1	NP_048532
PbCV-AR158	YP_001498312
PbCV-NY2A	P30320
PbCV-FR483	YP_001425655
AtCV1	YP_001427279

Table S2. Host range test of MpoV-44T.A, MpoV-44T.B and MpoV-44T lysate on 11 different *Micromonas* strains at four different temperatures. grey = lysed

Temperature (^{o}C)	Species	Strain	Origin	MpoV-		
				**44T**	**44T.A**	**44T.B**
3	*M. polaris*	RCC2258	Beaufort Sea	(grey)	(grey)	(grey)
	M. polaris	RCC2257	Beaufort Sea	(grey)	(grey)	(grey)
	M. polaris	NIOZ TX-01	Svalbard	(grey)	(grey)	(grey)
	M. commoda	LAC38	Oslofjord, Norway	(grey)	(grey)	(grey)
4	*M. polaris*	RCC4779	Svalbard			
	M. polaris	RCC4778	Svalbard			
	M. polaris	RCC4298	Svalbard			
	M. polaris	RCC2242	Bering Sea		(grey)	
7	*M. polaris*	RCC2258	Beaufort Sea	(grey)	(grey)	(grey)
	M. polaris	RCC2257	Beaufort Sea	(grey)	(grey)	(grey)
	M. polaris	RCC2246	Beaufort Sea	(grey)	(grey)	(grey)
	M. polaris	CCMP2099	Baffin Bay			
	M. pusilla	CCMP1545	English Channel	(grey)	(grey)	(grey)
	M. commoda	LAC38	Oslofjord, Norway	(grey)	(grey)	(grey)
15	*M. pusilla*	CCMP1545	English Channel			
	M. commoda	LAC38	Oslofjord, Norway	(grey)		(grey)

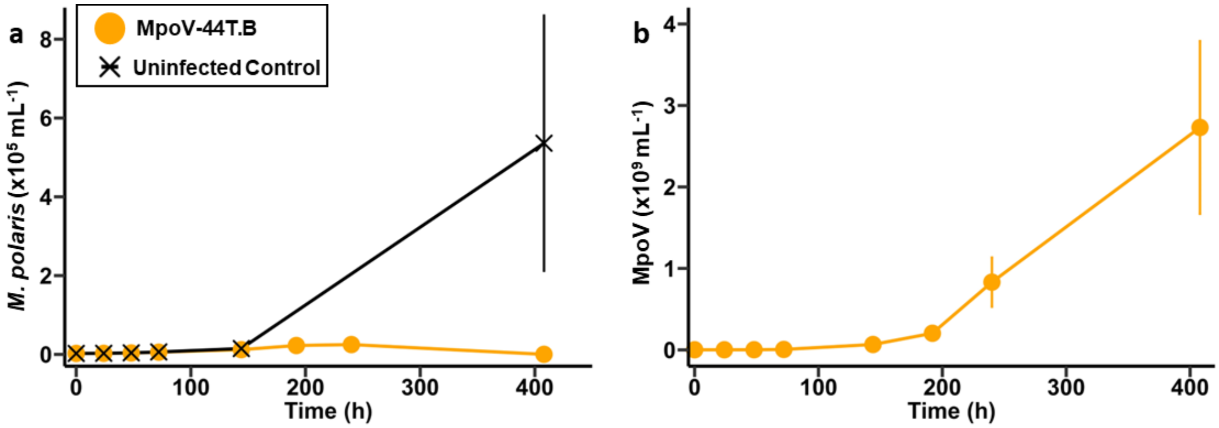

Fig. S1. Infection dynamics of MpoV-44T.B over the course of 408 hours (17 days) (a) *Micromonas polaris* and (b) MpoV particle dynamics obtained by flow cytometry. Results are shown as averages ± standard deviation (*n*=3).

Table S3. Screening of archived MpoV-44T lysates for presence of MpoV-44T.A and 44T.B using qPCR. The lysates originate from the MpoV-44T lysate isolated from Kongsfjorden in December 2006 (Maat *et al.*, 2017) and were maintained by monthly addition of 0.5 mL lysate to 4.5 mL of exponentially growing host. After host lysis, which takes around a week, lysates were stored at 4 °C. Black = present.

Date	MpoV-44T.A	MpoV-44T.B
03/11/2008		
01/08/2009	■	■
09/11/2009		
18/11/2009		
11/01/2010		
12/02/2010		
13/05/2010		
24/01/2012		
01/05/2012	■	■
01/05/2012		■
18/10/2016	■	■
18/10/2016	■	■
21/11/2016	■	■
01/02/2017	■	■
06/06/2018	■	■
09/10/2018	■	■
21/08/2018	■	■
20/03/2019	■	■
23/04/2019	■	■
09/09/2019	■	■
11/02/2020	■	■
26/02/2020	■	■
18/03/2020	■	■
06/05/2020	■	■
17/6/2020	■	■
30/10/2020	■	■
01/02/2021	■	■
01/05/2021	■	■
11/12/2021	■	■
02/03/2022	■	■
09/06/2022	■	■
21/7/2022	■	■
24/4/2023	■	■
11/10/2023	■	■
